# Association between glycosylated hemoglobin A1c and bone biochemical markers in type 2 diabetic postmenopausal women: a cross-sectional study

**DOI:** 10.1186/s12902-019-0357-4

**Published:** 2019-03-12

**Authors:** Lianzi Wang, Tao Li, Jiaqing Liu, Xian Wu, Huihui Wang, Xuemei Li, Enjun Xu, Qiuli Chen, Chuan Yan, Huimin Li, Yuanhong Xu, Wei Wei

**Affiliations:** 1 Department of Clinical Laboratory, The First Affiliated Hospital of Anhui Medical University, No. 218 Jixi Road, Hefei, 230032 Anhui China; 2 Institute of Clinical Pharmacology, Anhui Medical University, Key Laboratory of Anti-inflammatory and Immune Medicine, Ministry of Education, Anhui Collaborative Innovation Center of Anti-inflammatory and Immune Medicine, Anhui Anti-inflammatory and Immune Medicine innovation team, Hefei, 230032 China

**Keywords:** HbA1c, N-MID osteocalcin, PINP, PTH, β-CTX, 25(oh)D_3_

## Abstract

**Background:**

type 2 diabetes mellitus (T2DM) is a complicated disease that can affect bone health, but the change in bone biochemical markers caused by T2DM was controversial, so the aim of this study was to investigate whether there was a discrepancy in the levels of bone biochemical markers between postmenopausal women with T2DM and non-diabetic women and to explore the relationship between the level of glycosylated hemoglobin A1c (HbA1c) and bone biochemical markers in these subjects.

**Methods:**

A total of 237 type 2 diabetic postmenopausal women visiting the First Affiliated Hospital of Anhui Medical University from January 2017 to October 2018 and 93 healthy postmenopausal women were retrospectively enrolled. The differences in the levels of bone biochemical markers between patients and controls were analyzed by one-way ANOVA or chi-square test. The relationship between HbA1c and bone biochemical markers was analyzed by multivariate regression, forest plot and fitted curve.

**Results:**

Bone formation markers including N-MID osteocalcin and procollagen type 1 amino-terminal pro-peptide (PINP) were decreased in postmenopausal women with T2DM compared to controls (17.42 ± 9.50 vs 23.67 ± 7.58, *p* < 0.001; 48.47 ± 27.27 vs 65.86 ± 21.06, p < 0.001, respectively), but the bone resorption markers β-crossLaps (β-CTX) was no difference between the two groups (0.57 ± 0.28 vs 0.55 ± 0.21, *p* = 0.868). Multivariate regression showed that HbA1c was inversely associated with N-MID osteocalcin and PINP after adjusting for age, BMI, menopause^’^s years, diabetic duration, TC, TG, HDL-c, LDL-c, creatinine, UA and eGFR. The adjusted coefficients for N-MID osteocalcin and PINP per 1% HbA1c decrease were − 0.71 (− 1.19, − 0.22) and − 1.79 (− 3.30, − 0.28), respectively. A segmentation effect was seen in the fitted curve between HbA1c and β-CTX with an inflection point at 7.4% of HbA1c, the highest quartile of β-CTX (> = 0.74 ng/ml) showed a significantly negative with HbA1c. No significant association was seen between HbA1c and other biochemical markers.

**Conclusions:**

Our study found that bone formation was inhibited in postmenopausal women with T2DM, but bone resorption was not affected, and poor glycemic control was related to lower levels of bone formation, may increase the risk of bone fracture in postmenopausal women with T2DM.

## Background

Type 2 diabetes mellitus (T2DM), a complex multifactorial disease affecting sugar, fat and protein metabolism, has consistently been a global health problem with negative effects on mortality. It can also lead to a dysregulation in the handling of calcium, phosphorus and magnesium, thus leading to a series of complications such as cardiovascular disease, peripheral vascular disease, retinal disease, and neuropathy. Bone is also affected by T2DM. Osteoporosis is a long-term complication of T2DM that is prevalent in postmenopausal women, and previous studies have shown that risk of fractures was significantly higher in patients with T2DM than in those without T2DM; therefore, researchers have focused on the effect of diabetes on bone mineral density (BMD). BMD has been shown to be decreased or normal in T2DM patients in some studies [1–4], while other studies have reported that T2DM is associated with higher BMD [5]. Recently, increasingly more attention has been paid to the differences in bone metabolism in patients with diabetes mellitus. Bone biochemical markers reflect the bone turnover process and hence mirror bone resorption and formation processes [6]. Changes in bone turnover can be assessed by measuring serum levels of β-CTX, PINP, parathyroid hormone (PTH), N-MID osteocalcin and 25-hydroxyvitamin D_3_ (25(OH)D_3_), which have the advantage of reflecting alterations in bone formation and resorption.

However, studies on bone biochemical markers in T2DM have shown conflicting results, with the inhibition of bone formation being reported in most cases [7–9]; a recent survey indicated that there was no difference in bone turnover between Iranian postmenopausal women with and those without diabetes [10]. HbA1c is a stable marker that can characterizes dysglycemia more efficiently than fasting glucose in terms of long-term glucose regulation, Puar TH et al. noted that tight glycemic control (when HbA1c < 7%) was closely related with a greater risk of hip fracture in individuals being treated for T2DM [11]; in contrary, Schwartz et al. found no increase in the fall risk of patients with standard glydemic control [12]. However, little research has focused on the association between the level of HbA1c and bone biochemical markers. And given the large heterogeneity of these studies, we conducted these experiments to identify the differences between T2DM patients and controls and formulated the following hypothesis: glycosylated hemoglobin A1c levels affect the level of bone biochemical markers.

## Subjects and methods

### Subjects

This was a cross-sectional study that included patients from the First Affiliated Hospital of Anhui Medical University diagnosed with T2DM. Patients with the following diseases that influenced the bone metabolism ware excluded: hyperparathyroidism, hypercortisolism, osteoporosis, chronic liver disease, malignant tumor, Cushing’s syndrome, acromegaly, rheumatoid arthritis, thyroid disease, ankylosing spondylitis, chronic obstructive pulmonary disease and chronic kidney disease including diabetic nephropathy. Patients who had a history of taking estrogen, parathyroid hormone, glucocorticoids (more than 3 months), anti-seizure drugs, bisphosphonates was also excluded. A group of 237 postmenopausal women were ultimately included in this study. The average age of the subjects was 64.41 ± 9.23 years. Recruitment occurred from January 2017 to October 2018, in additional, postmenopausal women visited the hospital only for a health examination matched for age to T2DM with normal glucose tolerance from healthy examination center of the First Affiliated Hospital of Anhui Medical University were enrolled in our survey, according to the medical examination report, 93 controls meet our standard in the end, average age of the controls was 64.61 ± 7.62 years. T2DM was defined according to the updated and approved diagnostic criteria for diabetes of the American Diabetes association (ADA) released in 2017, when fasting plasma glucose was (FPG) ≥7 .0mmol/L and/or 2-h posted-glucose (PG) was ≥200 mg/dL (11.1mmol/L) during oral glucose tolerance test (OGTT) and/or HbA1c ≥6.5%; normal glucose tolerance was a status that fasting plasma glucose was (FPG) < 6 .1mmol/L and/or 2-h posted-glucose (PG) was < 7 .8mmol/L) during oral glucose tolerance test (OGTT). This research was approved by the Ethics Review Committee of the First Affiliated Hospital of Anhui Medical University (Reference number Quick-PJ2018-11-11).

### Biochemical measurements

#### Blood specimen collection

Blood samples were collected after fasting for at least 8 h. The samples were then centrifuged for 5 min at 3500 r/s within 2 h. The serum was then stored at − 20 °C and was subsequently used to measure the levels of bone biochemical markers, including β-CTX, PINP, PTH, N-MID osteocalcin and 25(OH)D_3_ and other variables, including total cholesterol (TC), triglycerides (TG), high-density lipoprotein-cholesterol (HDL-c), low-density lipoprotein-cholesterol (LDL-c), creatinine, uric acid (UA) and estimated glomerular filtration rate (eGFR).

#### Methods

The bone markers PTH, PINP, β-CTX, N-MID osteocalcin and 25(OH)D_3_ were measured using electrochemiluminescence immunoassay (Cobas 601, Roche Diagnostics), and glycosylated hemoglobin A1c (HbA1c) was measured by high-performance liquid chromatography (HPLC) using Bio-Rad D-10. Serum TC, TG, HDL-c, and LDL-c were detected by immunoturbidimetry. The within and between batch coefficients of variation were both < 8%. The laboratory was certified by ISO 15189.

The weight of the patients was measured with a calibrated balance-beam scale and height was measured using a stadiometer. Body Mass Index (BMI) was calculated using the formula weight divided by the square of height (kg/m^2^). The information containing patient^’^s age, BMI, menopause^’^s years, duration of T2DM, diabetic complications including microvascular complications(diabetic peripheral neuropathy (DPN) and diabetic retinopathy (DR)) and macrovascular complications (including atherosclerosis, hypertension), history of fracture, use of stain medication, and treatment type of hyperglycemia were collected.

### Statistical analysis

All continuous variables were expressed as the mean ± standard deviation(SD) or median (interquartile range), while categorical variables were described as a frequency or percentage. One-way ANOVA was used to compare continuous variables, and a chi-square test was used for categorical variables. Multiple regression analysis was used to estimate the independent association between HbA1c and bone biochemical markers while adjusting for potential confounders. Stratified and interaction analyses were implemented according to age (< 60 or ≥ 60 years), menopause years (< 10 or ≥ 10 years), BMI (< 24 or ≥ 24 kg/m^2^), diabetic duration (< 10 or ≥ 10 years), history of fracture (yes or no), microvascular complications (yes or no), macrovascular complications (yes or no), and treatment type (insulin, anti-diabetic drugs or combination therapy), use of stain medication(yes or no), the results were presented through forest plot. Spline smoothing of the generalized additive models was performed to explore the relationship between HbA1c and these outcomes.

All of the analyses were performed using the software R for statistical computing (http://www.R-project.org, The R Foundation) and the software Stata (version 14.1, Stata Corp). *P* < 0.05 was considered statistically significant.

## Results

### Comparison of bone biochemical markers between postmenopausal women with T2DM and the controls

Bone metabolism markers in T2DM patients and controls are shown in Table 1. Bone formation markers N-MID osteocalcin and PINP were significantly lower in postmenopausal women with T2DM than in the controls. Similarly, there was an obvious reduction in PTH and 25(OH)D_3_ in T2DM patients compared to controls. However, we were unable to find a significant difference in the bone resorption marker β-CTX between the diabetic group and the control group, the levels of blood lipids were also significantly different between the two groups.

**Table 1:** General characteristics of the T2DM and controls

	T2DM (*n* = 237)	Control (*n* = 93)	*P* value
age(years)	64.41 ± 9.23	64.61 ± 7.62	0.900
Menopause’s years(years)	13.77 ± 9.55	14.13 ± 13.21	0.783
Diabetic duration(years)	12.08 ± 8.64	None	NA
Fasting glucose(mmol/L)	NA	5.32 ± 0.60	NA
HbA1c	9.36 ± 2.35	5.67 ± 0.33	< 0.001
PINP(ng/ml)	48.47 ± 27.27	65.86 ± 21.06	< 0.001
PTH(pg/ml)	43.43 ± 30.05	50.34 ± 20.85	< 0.001
N-MID osteocalcin(ng/ml)	17.42 ± 9.50	23.67 ± 7.58	< 0.001
β-CTX(ng/ml)	0.57 ± 0.28	0.55 ± 0.21	0.868
25(OH)D_3_(ng/ml)	11.22 ± 6.83	18.39 ± 5.62	< 0.001
TC(mmol/L)	4.54 (3.79–5.31)	5.25(4.60,6.02)	< 0.001
TG(mmol/L)	1.45 (1.03–2.17)	1.21(0.95,1.67)	0.002
HDL-c(mmol/L)	1.16 (0.96–1.41)	1.58(1.34,1.82)	< 0.001
LDL-c(mmol/L)	2.67 (1.99–3.30)	3.23(2.46,3.85)	< 0.001
History of fracture, n (%)	17 (7.17%)	None	NA
Microvascular complications, n (%)	183 (77.22%)	None	NA
Macrovascular complications, n (%)	144 (60.76%)	None	NA
Treatment		None	NA
Insulin, n(%)	63 (26.58%)	None	NA
Oral anti-diabetic agents, n (%)	23 (9.70%)	None	NA
Insulin+Oral anti-diabetic agents, n (%)	151 (63.71%)	None	NA
Use of statin medication	112 (47.26%)	None	NA

Data were presented as number (percentage) for categorical data, (mean ± standard deviation) for parametrically distributed data or median (interquartile range) for nonparametrically distributed data. NA: not available

### General characteristics of postmenopausal patients with T2DM

A total of 214 postmenopausal patients with T2DM were enrolled in this research. The clinical information of the recruited patients was listed in Table 2. The mean age was 64.41 ± 9.23 years, mean menopause^’^s years was 13.77 ± 9.55 years, mean diabetic duration was 12.08 ± 8.64 years, and mean BMI was 12.08 ± 8.64 (kg/m2). Only 17 patients had a history of fracture; 183 patients were accompany with microvascular complications; 144 patients were suffered with macrovascular complications; 63 of 214 patients received insulin treatment only, 23 patients used anti-diabetic drugs only, 151 patients were using combination therapy(Table 2). The patients were divided into two groups according to the level of their serum HbA1c level (greater or less than 8.5%)(Table 3). A higher level of serum N-MID osteocalcin and PTH was observed in the HbA1c < 8.5 group (*p* < 0.05), but no significant difference in the level of serum PINP, β-CTX and 25(OH)D_3_ was observed. History of fracture, microvascular or macrovascula rcomplications and treatment strategy also had no difference between the HbA1c < 8.5% group and the HbA1c > 8.5% group, however, the use of statin medication was different obviously, moreover, individuals with higher HbA1c level were likely to have a higher levels of eGFR and TG but a lower levels of creatinine and HDL-c.

**Table 2:** General characteristics of the T2DM in postmenopausal women according to HbA1c more or lower than 8.5%

	HbA1c < 8.5%	HbA1c ≥ 8.5%	*P* value
N	103	134	
Age(years)	65.38 ± 9.36	63.67 ± 9.09	0.158
Menopause’s years(years)	14.32 ± 9.37	13.34 ± 9.71	0.436
Diabetic duration(years)	12.44 ± 8.20	11.81 ± 8.98	0.583
BMI(Kg/m^2^)	24.54 ± 3.74	24.21 ± 4.16	0.525
eGFR(mL/min/1. 73m^2^)	81.43 ± 24.98	92.40 ± 21.45	0.001
creatinine(μmol/L)	77.21 ± 43.50	63.02 ± 22.33	0.001
UA(mg/dL)	301.52 ± 96.58	278.51 ± 103.94	0.083
TC(mmol/L)	4.52 (3.84–5.16)	4.55 (3.76–5.41)	0.666
TG(mmol/L)	1.36 (1.02–1.82)	1.65 (1.10–2.42)	0.013
HDL-c(mmol/L)	1.22 (1.05–1.47)	1.11 (0.92–1.33)	0.007
LDL-c(mmol/L)	2.68 (2.04–3.19)	2.63 (1.98–3.42)	0.792
N-MID osteocalcin(ng/ml)	19.84 ± 10.71	15.56 ± 8.01	< 0.001
PINP(ng/ml)	51.71 ± 29.88	45.97 ± 24.90	0.153
β-CTX(ng/ml)	0.61 ± 0.31	0.53 ± 0.26	0.062
PTH(pg/ml)	49.09 ± 37.89	39.08 ± 21.38	0.021
25(OH)D_3_(ng/ml)	10.94 ± 6.92	11.44 ± 6.78	0.492
History of fracture,n (%)	6 (5.83%)	11 (8.21%)	0.614
Microvascular complications, n (%)	78 (75.73%)	105 (78.36%)	0.632
Macrovascular complications, n (%)	63 (61.17%)	81 (60.45%)	0.911
Treatment			
Insulin,n(%)	27 (26.21%)	36 (26.87%)	0.901
Oral anti-diabetic agents,n(%)	9 (8.74%)	14 (10.45%)	
Insulin+Oral anti-diabetic agents, n(%)	67 (65.05%)	84 (62.69%)	
Use of statin medication	37 (35.92%)	75 (55.97%)	0.002

Data were presented as number (percentage) for categorical data, (mean ± standard deviation) for parametrically distributed data or median (interquartile range) for nonparametrically distributed data

**Table 3:** Association between the level of HbA1c with bone biochemical markers in T2DM of postmenopausal women

	N-MID osteocalcin	PINP	β-CTX	PTH	25(OH)D_3_
	β(95%CI)	β(95%CI)	β(95%CI)	β(95%CI)	β(95%CI)
HbA1c					
Model1	−0.92 (−1.42, − 0.41)	−1.98 (−3.44, − 0.52)	− 0.01 (− 0.03, 0.00)	−1.29 (−2.91, 0.34)	0.05 (− 0.32, 0.42)
Model2	− 0.71 (− 1.19, − 0.22)	−1.79 (− 3.30, − 0.28)	−0.01 (− 0.03, 0.00)	−0.14 (− 1.49, 1.21)	−0.00 (− 0.39, 0.39)
Model3	− 0.71 (− 1.20, − 0.22)	− 1.80 (− 3.30, − 0.30)	−0.01 (− 0.03, 0.00)	−0.14 (− 1.50, 1.21)	−0.00 (− 0.39, 0.39)
Model4	− 0.71 (−1.20, − 0.22)	−1.85 (− 3.36, − 0.34)	− 0.01 (− 0.03, 0.01)	−0.13 (− 1.49, 1.24)	−0.02 (− 0.41, 0.37)
Model5	− 0.70 (− 1.20, − 0.20)	− 1.73 (− 3.27, − 0.20)	−0.01 (− 0.03, 0.01)	−0.10 (− 1.50, 1.29)	−0.10 (− 0.50, 0.30)

Values were based on linear regression models and reflect the difference and 95%CI for HbA1c, Model1 was non-adjusted, Model 2 was adjusted for age, BMI, menopause^’^s years, diabetic duration, TC, TG, HDL-c, LDL-c, creatinine, UA and eGFR. Model 3 was adjusted for variables in Model 2 and history of fracture (Yes or No). model 4 was adjusted for variables in Mode3, microvascular complications (Yes or No) and macrovascular complication (Yes or No). model 5 was adjusted for variables in Mode4, the treatment of T2DM (insulin, anti-diabetic agents or insulin+ anti-diabetic agents) and the use of statin medication(Yes or No)

### Association between HbA1c and the bone biochemical markers in postmenopausal women with T2DM

To investigate whether the bone biochemical markers levels in T2DM are associated with HbA1c, we performed a multivariate regression analysis after adjusting for different variables (Table 3). Linear relationships were observed between HbA1c and N-MID osteocalcin or PINP in all of the five adjusted models. In the unadjusted model (model 1), HbA1c increments were negatively correlated with N-MID osteocalcin and PINP, and each 1% HbA1c increment was associated with a lower level of N-MID osteocalcin with a β (95% CI) of − 0.92 (− 1.42, − 0.41), and a lower level of PINP with a β (95% CI) of − 1.98 (− 3.44, − 0.52), however, after adjusting for potential confounders (model 2, model 3, model 4 and model 5), the association between HbA1c and N-MID osteocalcin was attenuated but still present, the trend in the relationship between HbA1c and PINP was also weakened, the negative relationships between HbA1c vs N-MID osteocalcin and HbA1c vs PINP among model2, 3, 4 and 5 had little change. Nevertheless, we did not find a linear association between HbA1c and the other biochemical markers studied.

**Fig. 1:**
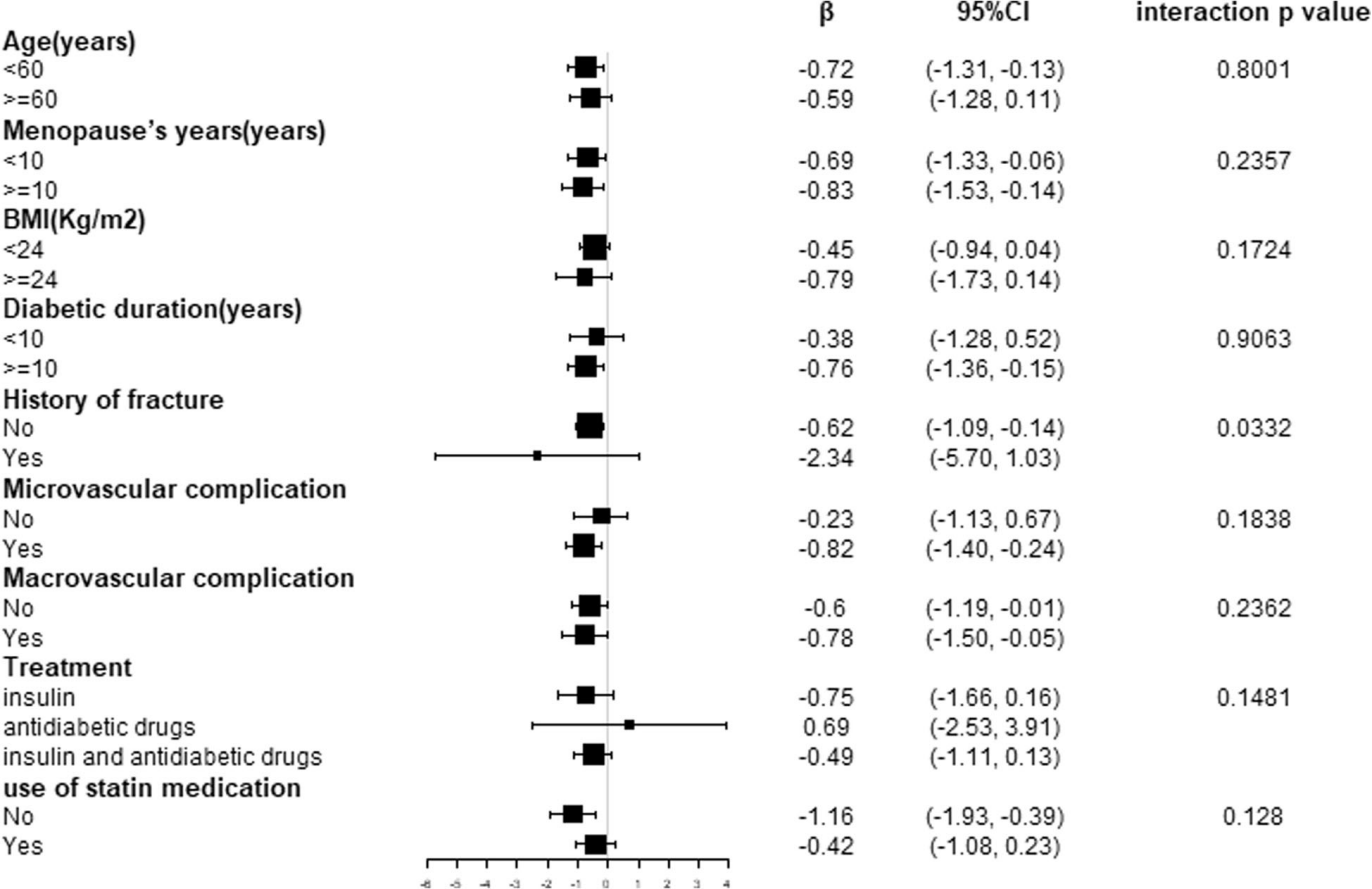
Associations of the N-MID osteocalcin with HbA1c in strata of age, menopause years, BMI, diabetic duration, history of fracture, microvascular complication, macrovascular complication, treatment, use of statin medication, and the interaction between subgroup.Data was adjusted for age, BMI, menopause^’^s years, diabetic duration, TC, TG, HDL-c, LDL-c, creatinine, UA and eGFR

**Fig. 2:**
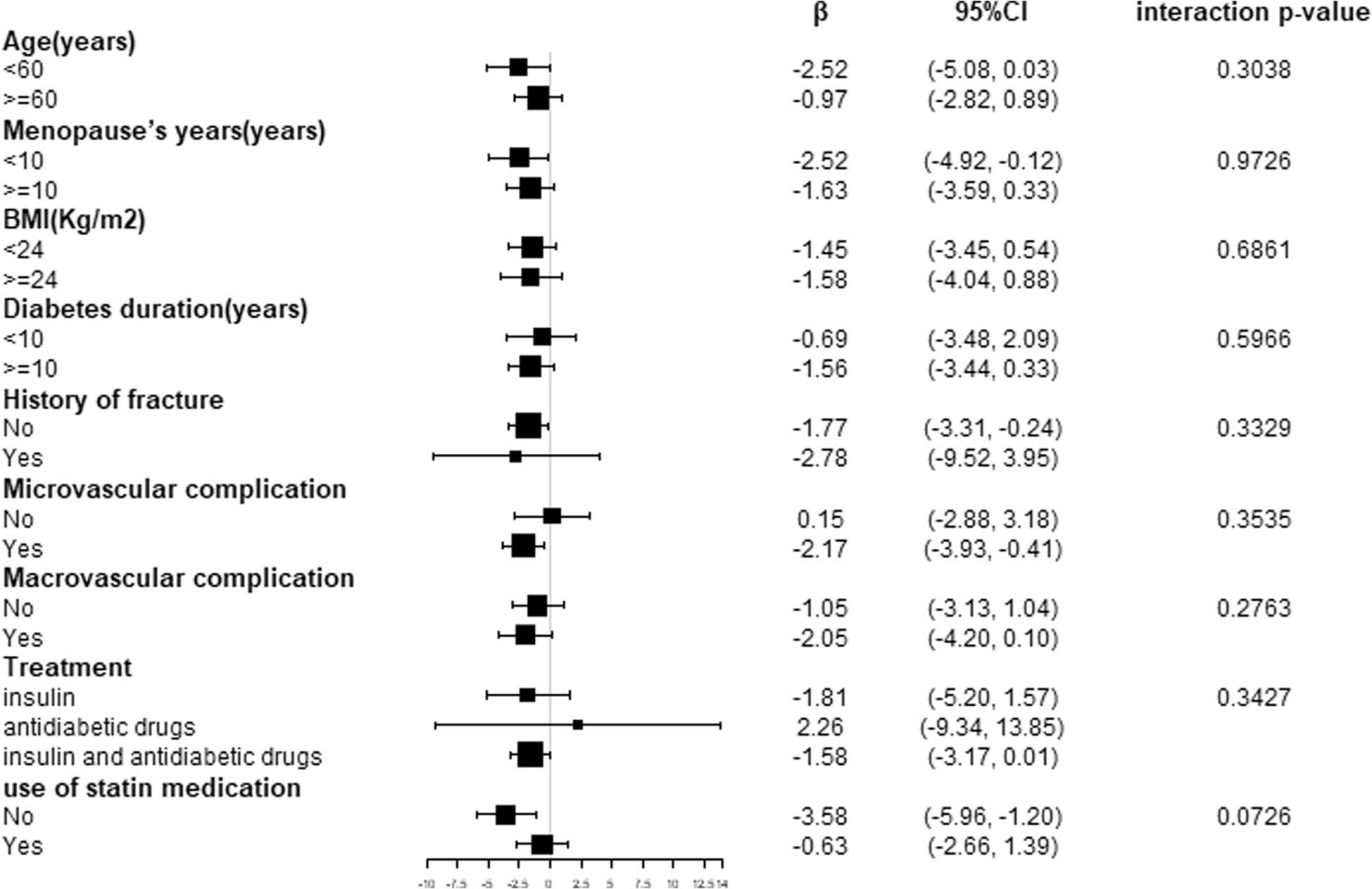
Associations of the P1NP with HbA1c in strata of age, menopause years, BMI, diabetic duration, history of fracture, microvascular complication, macrovascular complication, treatment, use of statin medication, and the interaction between subgroup.Data was adjusted for age, BMI, menopause^’^s years, diabetic duration, TC, TG, HDL-c, LDL-c, creatinine, UA and eGFR

Stratified and interaction analyses according to age (< 60 or ≥ 60 years), menopause^’^s years (< 10 or ≥ 10 years), and BMI (< 24 or ≥ 24 kg/m^2^) diabetic duration(< 10 or ≥ 10 years), history of fracture(yes or no), microvascular complications (yes or no), macrovascular complications (yes or no), treatment type (insulin, anti-diabetic drugs or combination therapy) and use of stain medication(yes or no) are shown in the forest plot (Fig. 1 and Fig. 2), In patients with a history of fracture, HbA1c was negatively correlated with N-MID osteocalcin with a β (95% CI) of − 2.34 (− 5.70, 1.03), while a β (95% CI) of − 0.62 (− 1.09, − 0.14), in the patients without a history of fracture, influence of HbA1c on bone biochemical markers in the two group was different significantly (interaction *p* value =0.033).

**Fig. 3:**
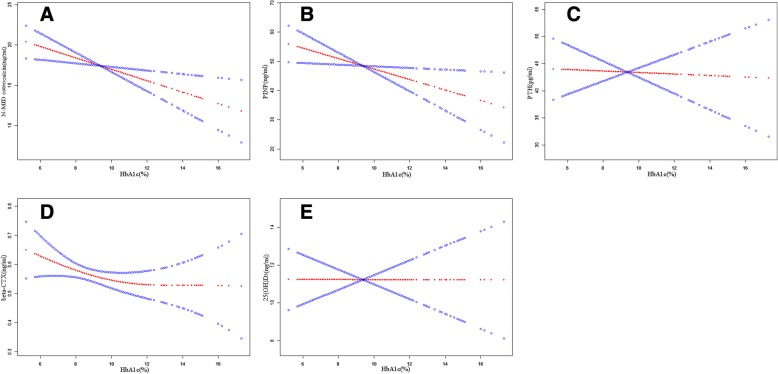
Smoothing splines of HbA1c and bone biochemical markers generated in generalized additive models. General associations between HbA1c and N-MID osteocalcin (**a**), and PICP (**b**); PTH (**c**), and β-CTX (**d**); 25(OH)D_3_ (**e**). Adjusted for age, BMI, menopause’s years, diabetic duration, TC, TG, HDL-c, LDL-c, creatinine, UA, and eGFR. The line of dark blue circles indicates 95% confidence intervals

As for the linear relationship between HbA1c and N-MID osteocalcin, and between HbA1c and PINP, we speculated that a nonlinear relationship existed between HbA1c and the other biochemical markers studied. We used smoothing splines generated in the generalized additive models to explore HbA1c and these outcomes (Fig. 3). A linear relationship was found between HbA1c and N-MID osteocalcin and between HbA1c and PINP with a statistically significant, which was consistent with the results of the multiple regression. Interestingly, a segmentation effect was observed between HbA1c and β-CTX in our investigation. A negative association was identified between HbA1c and β-CTX with a β (95% CI) of − 0.11 (− 0.22, − 0.01) when HbA1c was less than 7.4%, but no significant relation was observed with β-CTX when HbA1c was greater than 7.4% (Table 4). To testify this interesting segmentation effect, we quartered the level of β-CTX, and a multiple linear regression analysis was performed, as shown in Table 5, a significant negative relationship was shown between HbA1c and the highest quartile of β-CTX (> = 0.74 ng/ml), but no significant association with HbA1c when β-CTX was less than 0.74, which was nearly accordance with the fitted curve.

**Table 4:** Segmentation effect between HbA1c and β-CTX

	β-CTX	
	β(95%CI)	*P* value
HbA1c		
<=7.4%	−0.11 (− 0.22, − 0.01)	0.0325
> 7.4	−0.00 (− 0.02, 0.02)	0.8073

Data was adjusted for age, BMI, menopause^’^s years, diabetic duration, TC, TG, HDL-c, LDL-c, creatinine, UA and eGFR

**Table 5:** Association of β-CTX quartile as ordinal variables with HbA1c in multivariate regression

	β-CTX			
	Q1	Q2	Q3	Q4
Non-adjusted	−0.00 (− 0.01, 0.01)	0.00 (− 0.00, 0.01)	0.01 (− 0.00, 0.01)	−0.03 (− 0.04, − 0.01)
adjusted	−0.00 (− 0.01, 0.01)	0.00 (− 0.00, 0.01)	0.01 (− 0.00, 0.01)	−0.03 (− 0.05, − 0.01)

Adjusted variables: age, BMI, menopause^’^s years, diabetic duration, TC, TG, HDL-c, LDL-c, creatinine, UA and eGFR

Q1: <=0.35 ng/ml; Q2: 0.36–0.51 ng/ml; Q3: 0.52–0.73 ng/ml; Q4: > = 0.74 ng/ml

## Discussion

Clearly, glucose metabolism is closely related to bone metabolism, as bone biochemical markers in patients with diabetes were affected by many factors, such as the type of diabetes, the presence of complications, history of fractures, the treatment type and so on. However, how T2DM affects bone metabolism in postmenopausal women remains unclear. Studies on bone turnover markers in type 2 diabetic patients have reported conflicting results, and a consensus has not yet been reached. In this investigation, we explored the association between bone turnover and type 2 diabetes in postmenopausal women.

In this research, we found that the bone formation markers N-MID osteocalcin and PINP were lower in postmenopausal women with T2DM than in the controls, in agreement with most studies [7–9]. The inhibition of bone formation in T2DM may be due to the accumulation of advanced glycation end-products in the organic bone matrix, which may interfere with normal osteoblast function [13]. Furthermore, hyperglycemia may have a direct influence on bone cells by increasing the expression of sclerostin in osteocytes to inhibit bone formation [14].

At the same time, the bone resorption marker β-CTX did not show any discrepancies between diabetic patients and controls in our investigation. β-CTX was a confusing and controversial biochemical marker in T2DM all the time. Consistently with our results, β-CTX had no differ in T2DM patients and non-diabetic patients in some studies [15, 16], other bone resorption indices including bone surface and osteoclast number had no difference between T2DM and controls [17], indicating a suppression of bone formation in T2DM but no alteration in bone resorption. Other studies have shown conflicting results-that there is a lower level of β-CTX in patients with T2DM [18, 19]. However, we performed a univariate analysis only to analyze the discrepancy of bone markers between the two groups, thus, the results of studies was varied may be explained by differences in metabolic status, diabetic duration, and treatment type in patients with T2DM.

Among postmenopausal women with T2DM, we found N-MID osteocalcin and PINP was negatively correlated with HbA1c in type 2 diabetes with or without adjustment, indicating that a poor glycemic control may be associated with a reduction of bone formation. After adjusting for the variables we collected, the opposite relationship was weakened, interaction analyses in stratified subgroup showed age, menopause years, BMI, diabetic duration, history of fracture, microvascular complication, macrovascular complication and treatment of T2DM did not influenced the relation between HbA1c and PINP. However a stratification analysis according to history of fracture(Yes or No) conducted showing a significant difference of N-MID osteocalcin in the two subgroups based on HbA1c, which means HbA1c does not independently affect the level of N-MID osteocalcin, but is affected by a history of fractures. Some previous reports detected insulin treatment may not affected the bone biochemical markers, metformin treatments could decrease the bone biochemical markers [20, 21]; nevertheless, Dyah Purnamasari indicated insulin and metformin didn^’^t alter bone metabolism [8]. A study showed low serum UA concentration may be associated with lower BMD values [22], a population-based study of 3028 older older women indicated statin use was related with lower cortical porosity [23], the effect of diabetic complications on bone metabolism remains unknown, so we need to adjust these confounds in our study, and further research should pay more attention to the effect of renal function, anti-diabetic agents and diabetic complication on bone metabolism.

In a type 1 diabetic mouse model, Botolin et al. [24] detected that the function of osteoblasts was inhibited by hyperglycemia. This effect of hyperglycemia may be similar in humans, and our results suggest a dysfunction in osteoblasts in diabetic patients. Targeted knock out of the osteocalcin gene (Ocn−/−) in mice resulted in the development of glucose intolerance and a decrease in the number of pancreatic beta cells, while OC favored β-cell proliferation, insulin secretion, and sensitivity through adiponectin [25]. The wnt/β-catenin signaling pathway is also involved in glucose metabolism and affects the level of OC [26]. The association between the levels of HbA1c and its influence on OC through wnt/β-catenin signaling needs to be explored further.

A higher level of HbA1c induces insulin secretion and hypoinsulinemia inhibits the process of bone formation, and PINP was directly associated with insulin resistance, indicating that HbA1c affects insulin resistance and insulin sensitivity. Reactive oxygen species (ROS) was increased in diabetes by a variety of mechanisms including increased AGEs [27]. Therefore, we hypothesized that a higher level of HbA1c promotes the apoptosis of osteoblast and induces PINP through the production of ROS.

An interesting result in our study is the identification of a segmentation effect between HbA1c and β-CTX; an inverse relationship between HbA1c and β-CTX was found when HbA1c was less than 7.4%, but no association was noted when HbA1c was greater than 7.4%. When quartered the level of β-CTX, the highest quartile of β-CTX (> 0.74 ng/ml) showed a significantly negative with HbA1c. Recently, a study involving people with a normal glucose tolerance found that β-CTX was positively associated with HbA1c [28]. Ogata, Makiko et al. discovered that the CTX-1 level was significantly and negatively correlated with the patients initial HbA1c level and average HbA1c levels for the previous 6 month [29]. No relationship with β-CTX was has been noted in recent year [30]. Otherwise, β-CTX may be a predictor of fracture risk, a higher level of β-CTX may increase the risk of fracture, the large heterogeneity among studies could be caused by differences in patients^’^ characteristics indicated in a systematic review [20]. Kaori Kitaoka^’^s research may help us to interpret this phenomena, when HbA1c increased from 6.93 to 7.46%, DN progression was significantly accelerated in patients with T2DM, resulting in an increase in β-CTX exclusion [31]. Besides, a former report discovered the highest quartile of β-CTX was observably related with a rapid reduction of cortical bone loss [32], and a tight glycemic control (when HbA1c < 7%) was closely related with greater risk of hip fracture in individuals being treated for T2DM were all helpful to explain our results, however additional investigation involving a large clinical prospective survey are required to explore the significance of β-CTX in the development of T2DM.

Although many confounders was adjusted in our research, there are also some limitations. First, our sample size was not large enough. Second, the cross-sectional survey used in this study limited our ability to identify etiological factors, and since our survey was focused on postmenopausal women suffering from T2DM, the findings herein reported may not suitable for men. Third, we were unable to obtain all of the information regarding all the subjects involved in this study including BMD and risk factors for fracture in order to explore their relationship with HbA1c, bone biochemical markers and BMD. Fourth, detail information regarding the anti-diabetic medications taken by the subjects was not available in this study, since it has been shown that the level of bone biochemical markers was also influenced by different kinds of hypoglycemic drugs.

## Conclusions

Bone formation was inhibited in postmenopausal women with T2DM, but no alteration of the bone resorption marker β-CTX was observed compared to controls. HbA1c was inversely related to N-MID osteocalcin and PINP. Therefore, a poor glycemic control may aggravate bone loss, increasing the risk of fracture in patients with T2DM.

## Abbreviations

25(OH)D_3_: 25-hydroxyvitamin D_3_
ADA: American Diabetes Association
BMD: Bone mineral density
BMI: Body Mass Index
DN: Nephropathy
DPN: Diabetic peripheral neuropathy
DR: Diabetic retinopathy
eGFR: estimated glomerular filtration rate
HbA1c: Glycosylated hemoglobin A1c
HDL-c: High-density lipoprotein-cholesterol
HPLC: High-performance liquid chromatography
LDL-c: Low-density lipoprotein- cholesterol
OC: Osteocalcin
PINP: Procollagen type 1 amino-terminal pro-peptide
PTH: Parathyroid hormone
SD: Standard deviation
T2DM: Type 2 diabetes mellitus
TC: Total cholesterol
TG: Triglycerides
UA: Uric acid; creatinine
β-CTX: β-crossLaps
